# Web-Based Self-Management for Patients With Lymphoma: Assessment of the Reach of Intervention of a Randomized Controlled Trial

**DOI:** 10.2196/17018

**Published:** 2020-05-14

**Authors:** Lindy P J Arts, Simone Oerlemans, Eduardus F M Posthuma, Djamila E Issa, Margriet Oosterveld, René van der Griend, Marten R Nijziel, Lonneke V van de Poll-Franse

**Affiliations:** 1 Netherlands Comprehensive Cancer Organisation Utrecht Netherlands; 2 Center of Research on Psychology in Somatic Diseases Department of Medical and Clinical Psychology Tilburg University Tilburg Netherlands; 3 Department of Internal Medicine Reinier de Graaf Hospital Delft Netherlands; 4 Department of Internal Medicine Jeroen Bosch Hospital 's-Hertogenbosch Netherlands; 5 Department of Internal Medicine Canisius-Wilhelmina Hospital Nijmegen Netherlands; 6 Department of Internal Medicine Diakonessenhuis Utrecht/Zeist Netherlands; 7 Department of Internal Medicine Catharina Hospital Eindhoven Netherlands; 8 Division of Psychosocial Research and Epidemiology Netherlands Cancer Institute Amsterdam Netherlands

**Keywords:** reach, uptake, participation, web-based intervention, pragmatic, randomized controlled trial, population-based registry, lymphoma

## Abstract

**Background:**

Randomized controlled trials (RCTs) often provide accurate estimates of the internal validity of an intervention but lack information on external validity (generalizability). We conducted an RCT on the effectiveness of a self-management intervention among patients with lymphoma in a population-based setting.

**Objective:**

The objectives of the current study were to describe the proportion of RCT participants compared to all patients invited to participate, and compare sociodemographic and clinical characteristics of RCT participants with all respondents, all patients invited to participate, and all patients selected from the Netherlands Cancer Registry (NCR) to determine the reach of the intervention. An additional objective was to assess differences on RCT outcome variables between RCT and paper respondents.

**Methods:**

Patients with lymphoma or chronic lymphocytic leukemia ≥18 years old at diagnosis from 13 hospitals in the Netherlands were selected from the population-based NCR, which routinely collects data on sociodemographic and clinical characteristics. Eligible patients were invited to participate in an RCT and complete a questionnaire. Web-based completion determined RCT enrollment, whereas paper respondents were followed observationally.

**Results:**

A total of 1193 patients were selected from the NCR, 892 (74.77%) of whom were invited to participate in the trial by their hematologist after verifying eligibility. Among those invited, 25.4% (227/892) completed the web-based questionnaire and were enrolled in the RCT. The RCT participants were younger and there was a higher proportion of men than nonparticipants (*P*<.001). In addition, 25.7% (229/892) of those invited opted to participate in the paper-based observational follow-up study. Compared with paper respondents, RCT participants were younger (*P*<.001), with a higher proportion of men (*P*=.002), and had higher education levels (*P*=.02). RCT participants more often wanted to receive all available information on their disease (*P*<.001), whereas paper respondents reported higher levels of emotional distress (*P*=.009).

**Conclusions:**

From a population-based sample of eligible patients, the participation rate in the RCT was approximately 25%. RCT participants may not be representative of the target population because of different sociodemographic and clinical characteristics. Since RCT participants represent a minority of the target population, RCT results should be interpreted with caution as patients in the RCT may be those least in need of a self-management intervention.

**Trial Registration:**

Netherlands Trial Register NTR5953; https://www.trialregister.nl/trial/5790

## Introduction

Randomized controlled trials (RCTs) are widely considered to be the gold standard for evaluating the effects of an intervention in behavioral and psycho-oncological research [1,2]. In contrast to the effects of interventions that are most often examined extensively in RCTs [3], much less attention has been paid to the proportion of patients who participate in these interventions and whether those who choose to participate are representative of the target population in terms of sociodemographic and clinical characteristics [2,4]. Thus, RCTs often provide accurate estimates of the internal validity (ie, effect of an intervention for the sample enrolled in the RCT), but do not typically provide information about the external validity or generalizability (ie, effect of an intervention in the target population) [5-7].

The reach of an RCT provides information on the absolute number, proportion, and representativeness of the sample that participates in the trial [8]. The absolute number and proportion of RCT participants are relatively easy to assess and are therefore most often reported. However, few studies report the representativeness of the sample enrolled in an RCT, which is a much more challenging metric to assess [8,9] since it requires sociodemographic information, and preferable psychosocial, clinical, or case mix information, on RCT participants as well as nonparticipants. It is particularly challenging to collect information on nonparticipants who typically do not consent to be included in the research [8].

Interventions with promising effects in RCTs have been implemented in daily practice without specific knowledge of the generalizability of the results. Therefore, more attention should be paid to providing information related to the representativeness of the sample enrolled in an RCT. Lack of representativeness may occur as a result of inadequate selection procedures (ie, sampling bias) or when the probability of nonparticipation in the study is related to the object of research (ie, nonresponse bias) [10,11].

To fill this gap, the aim of the current study was to address the reach of a web-based self-management intervention within the context of the Lymphoma InterVEntion (LIVE) trial (Netherlands Trial Register NTR5953), whose objectives have been described elsewhere [12]. For the LIVE trial, patients were selected from the population-based Netherlands Cancer Registry (NCR) that routinely collects data on sociodemographic and clinical characteristics. This can provide unique insight into the characteristic differences between RCT participants and nonparticipants to estimate the reach of this intervention. The primary objectives were to (1) describe the proportion of RCT participants compared to all patients invited to participate, and (2) compare sociodemographic and clinical characteristics of RCT participants with those of all respondents (ie, patients who completed a web-based or paper questionnaire), all patients invited to participate, and all patients selected from the NCR. In addition, as patients had the option of completing a web-based questionnaire (ie, enrollment in the RCT) or a paper-based questionnaire (ie, no enrollment in the RCT; observational cohort), a secondary objective was to assess baseline differences in psychological distress, self-management skills, and satisfaction with information provision (ie, RCT outcome variables) between the two groups.

## Methods

### Study Design

Baseline data were collected from an RCT embedded in a population-based registry [13] as an observational cross-sectional dataset without information on the effectiveness of the intervention. In short, the LIVE trial examines the effectiveness of feedback on patient-reported outcomes and a web-based self-management intervention on self-management skills, satisfaction with information provision, and psychological distress among patients with lymphoma [12].

### Participants and Recruitment Procedure

From October 2016 to February 2019, patients who were diagnosed with lymphoma, including Hodgkin lymphoma, nonHodgkin lymphoma, or chronic lymphocytic leukemia (CLL) as defined by the International Classification of Diseases for Oncology-3 codes [14], from 13 hospitals in the Netherlands were selected for participation via the NCR. The NCR registers all patients newly diagnosed with cancer in the Netherlands within the first year after diagnosis and routinely collects detailed data on sociodemographic and clinical characteristics (eg, patient age and sex, date of cancer diagnosis, cancer type, and primary treatment). Patients had to be 18 years or older at the time of study invitation. Treating hematologists were asked to verify the patients’ eligibility for the study and to exclude patients who were deceased, had severe psychopathology, were too ill, were not able to complete a questionnaire in Dutch, or had severe cognitive impairment. All eligible patients were invited by mail to participate by their own hematologist. Patients had the option to complete a web-based or paper-based questionnaire. Patients were informed that completion of the web-based questionnaire automatically resulted in enrollment in the RCT with randomization to one of the study arms, whereas completion of a paper questionnaire resulted in participation in the observational Patient Reported Outcomes Following Initial treatment and Long term Evaluation of Survivorship (PROFILES) registry [13] but not enrollment in the RCT. To address the primary objective of the current study—describing the proportion of RCT participants compared to all patients invited to participate—paper respondents were assessed as nonparticipants as they did not participate in the RCT.

### Measures

#### Sociodemographic and Clinical Measures

Sociodemographic characteristics (age and sex) and detailed clinical information (date of diagnosis, cancer type, primary treatment) were available from the NCR. Information on education level and marital status was assessed from the questionnaire (data only available for respondents).

Comorbidities at the time of questionnaire completion were assessed using an adapted version of the self-administered comorbidity questionnaire [15]. Patients were asked to identify comorbidities present within the past 12 months, including heart disease, hypertension, arthritis, stroke, lung disease, diabetes, stomach disease, kidney disease, liver disease, anemia, thyroid disease, and rheumatoid arthritis. Positive responses were summed to a total score ranging from 0 to 12 (data only available for respondents).

#### Personality Traits

Personality traits were assessed with the Big Five Inventory, a 44-item inventory designed to measure the Big Five dimensions of personality: extraversion, neuroticism, conscientiousness, agreeableness, and openness to experience [16]. Each item was scored on a 5-point scale. Scale scores were obtained by averaging all items for each domain ranging from 0 to 5. Each trait is assumed to represent a continuum from high to low on the specific attribute and is partnered with a trait on the opposite pole of the spectrum [17,18].

#### Information Preferences

One question from an adapted version of the Information Satisfaction Questionnaire was used to measure information preferences [19]. Patients had to categorize themselves into one of three groups: those who would like (1) all available information, (2) only positive information about the illness, and (3) only limited information. Patients were further asked whether they use the internet (yes/no).

#### Psychological Distress, Self-Management Skills, and Satisfaction With Information Provision

Primary outcomes to assess the effectiveness of the intervention were psychological distress, self-management skills, and satisfaction with information provision.

Psychological distress was assessed with the 14-item Hospital Anxiety and Depression Scale [20]. Each item is rated on a 4-point scale from 0 to 3. The total score was obtained by adding all item scores and ranged from 0 to 42, in which higher scores indicate higher levels of psychological distress [21]. Patients with a Hospital Anxiety and Depression Scale sum score ≥13 were categorized as “psychologically distressed” [22].

Self-management skills were assessed with the Health Education Impact Questionnaire (heiQ) that contains 40 items across eight scales: positive and active engagement in life, health-directed activities, skill and technique acquisition, constructive attitudes and approaches, self-monitoring and insight, health service navigation, social integration and support, and emotional distress [23]. Each item is scored on a 4-point scale. Scale scores were obtained by averaging all items for each domain and ranged from 1 to 4. Higher scores indicate better status or self-management, except for emotional distress in which higher scores indicate greater distress [23].

Satisfaction with overall information provision was assessed with one item from an adapted version of the Information Satisfaction Questionnaire [19]. Patients were asked to rate their level of satisfaction for overall information provision on a scale from 1 (“very unsatisfied”) to 5 (“very satisfied”).

### Statistical Analyses

The proportion of RCT participants (ie, participation rate) was calculated by dividing the number of patients who were enrolled in the RCT by the total number of eligible patients who were invited to participate. Sociodemographic and clinical characteristics of RCT participants were compared with those of all respondents (ie, patients who completed a web-based or paper questionnaire), all patients invited to participate, and all patients selected from the population-based NCR. In addition, personality traits and information preferences of RCT participants were compared with those of all respondents. Differences on sociodemographic and clinical characteristics between RCT participants and nonparticipants were compared using analysis of variance for continuous variables and Chi-square tests for categorical variables.

Differences in baseline psychological distress, self-management skills, and satisfaction with information provision (ie, RCT outcome variables) between RCT participants and paper respondents were compared using analysis of variance for continuous variables and Chi-square tests for categorical variables. All statistical analyses were performed with SAS version 9.4 (Cary, NC, USA). *P*≤.05 indicated statistically significant differences.

## Results

### Patients Selected From the Netherlands Cancer Registry

**Figure 1:**
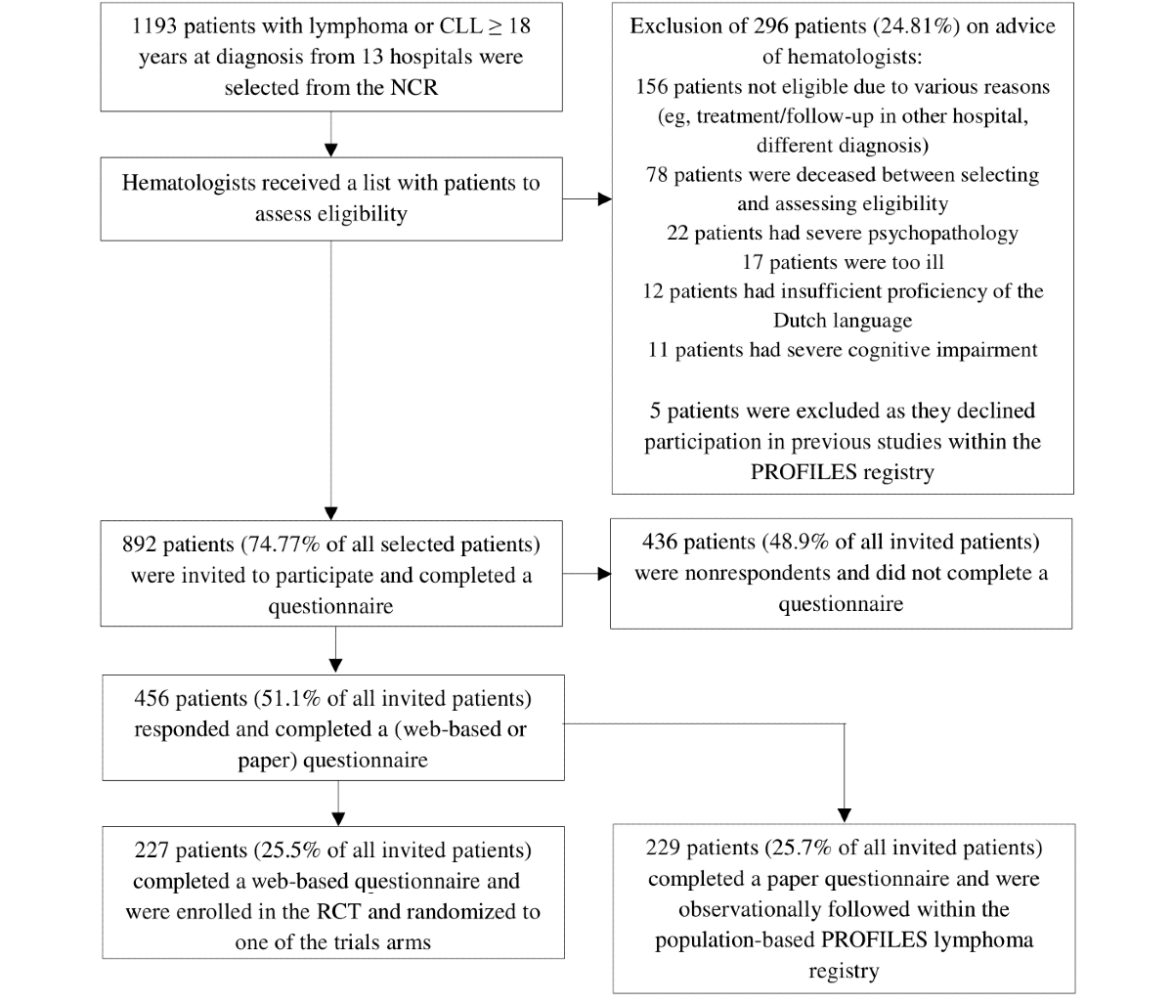
Flowchart of the data collection process. CLL: chronic lymphocytic leukemia; NCR: Netherlands Cancer Registry; RCT: randomized controlled trial; PROFILES: Patient Reported Outcomes Following Initial treatment and Long term Evaluation of Survivorship.

As shown in Figure 1, a total of 1193 patients with lymphoma or CLL who were ≥18 years old at diagnosis from 13 hospitals were selected from the population-based NCR. The basic characteristics of the invited patients are summarized in Table 1. The majority of patients were men, were diagnosed with high-grade nonHodgkin lymphoma, and were actively being treated, with chemotherapy as the most common treatment.

**Table 1:** Baseline characteristics of patients selected from the Netherlands Cancer Registry (NCR) according to participation^a^.

Characteristic				Patients selected from the NCR (N=1193)		Patients invited to participate (n=892)		Respondents (n=456)		RCT^b^ participants (n=227)
**Sociodemographic characteristics, n (%)**										
	Age at time of survey (years), mean (SD)		64.7 (15.6)		64.1 (15.3)		64.5 (13.5)		60.7 (13.4)	
	**Sex, n (%)**									
		Male	725 (60.8)		537 (60.2)		291 (63.8)		161 (70.9)	
		Female	468 (39.2)		355 (39.8)		165 (36.2)		66 (29.1)	
	**Education, n (%)**									
		Low^c^	N/A^d^		N/A		33 (7.2)		6 (2.6)	
		Medium^e^	N/A		N/A		260 (57.0)		106 (46.7)	
		High^f^	N/A		N/A		159 (34.9)		114 (50.2)	
	Partner (yes), n (%)		N/A		N/A		355 (77.9)		190 (83.7)	
**Clinical characteristics**										
	Months since diagnosis, mean (SD)		13.9 (3.5)		14.0 (3.4)		14.2 (3.3)		14.0 (3.2)	
	**Cancer type, n (%)**									
		HL^g^	120 (10.1)		102 (11.4)		46 (10.1)		27 (11.9)	
		NHL-HG^h^	676 (56.7)		484 (54.3)		260 (57.0)		125 (55.1)	
		NHL-LG^i^	280 (23.5)		224 (25.1)		114 (25.0)		56 (24.7)	
		CLL^j^	116 (9.7)		82 (9.2)		36 (7.9)		19 (8.4)	
	**Primary treatment, n (%)**									
		Active surveillance	315 (26.4)		252 (28.3)		113 (24.8)		53 (23.3)	
		CT^k^ alone	522 (43.8)		405 (45.4)		222 (48.7)		109 (48.0)	
		RT^l^ alone	80 (6.7)		70 (7.8)		31 (6.8)		11 (4.8)	
		CT + RT	103 (8.6)		90 (10.1)		51 (11.2)		29 (12.8)	
		SCT^m^/CT/ RT	30 (2.5)		25 (2.8)		20 (4.4)		16 (7.0)	
		Other	56 (4.7)		39 (4.4)		16 (3.5)		7 (3.1)	
		Unknown	87 (7.3)		11 (1.2)		3 (0.7)		2 (0.9)	
	Number of self-reported comorbidities, mean (SD)		N/A		N/A		1.3 (1.2)		1.1 (1.1)	
**Personality traits, mean (SD)**										
	Openness		N/A		N/A		3.4 (0.6)		3.5 (0.6)	
	Conscientiousness		N/A		N/A		3.7 (0.5)		3.7 (0.4)	
	Extraversion		N/A		N/A		3.5 (0.6)		3.5 (0.6)	
	Agreeableness		N/A		N/A		3.8 (0.5)		3.8 (0.4)	
	Neuroticism		N/A		N/A		2.5 (0.7)		2.4 (0.6)	
**Information preferences, n (%)**										
	All available information		N/A		N/A		211 (46.3)		126 (55.5)	
	Only positive information		N/A		N/A		65 (9.9)		23 (10.1)	
	Limited information		N/A		N/A		170 (37.3)		78 (34.4)	
Internet use (yes)			N/A		N/A		370 (81.1)		220 (96.9)	

^a^See Figure 1 for the flowchart of patient selection. The groups in columns are not mutually exclusive; RCT participants are also included in the groups of the other 3 columns.

^b^RCT: randomized controlled trial.

^c^Low education level: none/primary school.

^d^N/A: data not available.

^e^Medium education level: lower general secondary education/vocational training.

^f^High education level: preuniversity education/high-level vocational training/university.

^g^HL: Hodgkin lymphoma.

^h^NHL-HG: high-grade nonHodgkin lymphoma.

^i^NHL-LG: low-grade nonHodgkin lymphoma.

^j^CLL: chronic lymphocytic leukemia.

^k^CT: chemotherapy.

^l^RT: radiotherapy.

^m^SCT: stem cell transplantation.

### Patients Invited to Participate

A flowchart of the patient selection process is shown in Figure 1. Of all selected patients, 24.81% (296/1193) were excluded after verifying eligibility by their treating hematologists for the following reasons: deceased, severe psychopathology, too ill, insufficient proficiency of the Dutch language, and cognitive impairment. In addition, 156 patients were not eligible for other reasons, including 36 patients who received treatment or follow up in another hospital and 20 patients for whom the ultimate diagnosis did not meet our inclusion criteria (eg, myelodysplastic syndrome, acute lymphoblastic leukemia). The remaining 100 patients were excluded by the hematologists for unknown reasons. Furthermore, 5 patients were excluded as they declined participation in previous studies within the PROFILES registry. After exclusion of these patients, 892 patients (74.77%) were invited to participate and completed a questionnaire. Patients invited to participate did not significantly differ from all patients selected from the NCR in terms of age (*P*=.38) and sex (*P*=.07) (Table 1).

### Respondents

Among the 892 invited patients, 456 patients (51.1%) responded and completed either a web-based or paper questionnaire. The mean age of all respondents (Table 1) was comparable with that of nonrespondents (63.8 years, *P*=.43), and the majority of the respondents were also men. Respondents did not differ from all patients selected from the NCR in terms of age (*P*=.81) and sex (*P*=.26). Respondents were more often actively treated than nonrespondents (75% vs 66%, *P*=.01). Half of the respondents (229/456, 50.2%) completed a paper questionnaire, whereas the other half (227/456, 49.8%) completed a web-based questionnaire and were enrolled in the RCT. Nearly half of the respondents stated that they would like to receive all available information, with a lower proportion preferring limited information, and even less indicating that they would like to receive only positive information about the illness (Table 1). Approximately 82% of all respondents reported using the internet.

### Randomized Controlled Trial Participants

A quarter of all invited patients (227/892) participated by completing a web-based questionnaire, which resulted in a participation rate of the RCT of 25.4%. The mean age of RCT participants was slightly lower than that of nonparticipants (ie, nonrespondents and paper respondents (n=665, 65.3 years, *P*<.001) with a slightly higher mean time since diagnosis, and comprised a higher proportion of men (70.9%, 161/227 vs 57.0%, 379/665, *P*<.001). The proportion of patients who were actively treated were comparable between RCT participants and nonparticipants (75.8%, 172/227 vs 69.0%, 459/665, *P*=.13). In addition, RCT participants were significantly younger than respondents, patients invited to participate, and patients selected from the NCR (all *P*<.001). Furthermore, there was a higher proportion of men among RCT participants compared with all patients invited to participate and all patients selected from the NCR (both *P*<.001).

### Randomized Controlled Trial Participants Versus Paper Participants

RCT participants were younger than paper respondents (60.7 vs 68.3 years, *P*<.001). In addition, RCT participants were more often male (71% vs 57%, *P*=.002), more highly educated (50% vs 20%, *P<*.001), and more often had a partner (84% vs 75%, *P*=.02). No significant differences were found between RCT and paper respondents regarding cancer type or primary treatment (*P*=.54 and *P*=.06, respectively). RCT participants also reported fewer comorbidities than paper respondents (1.1 vs 1.4, *P*=.02).

Concerning personality traits, RCT participants had lower scores on neuroticism (2.4 vs 2.6, *P*=.003) and higher scores on openness to experience (3.5 vs 3.4, *P*=.002) than paper respondents, although effect sizes were small (Cohen *d*=0.29 and 0.28, respectively). With respect to information preferences, the majority of RCT participants stated a preference for receiving all available information, whereas only 38.9% (89/229) of paper respondents indicated this preference (*P*=.001). Conversely, paper respondents more often preferred receiving limited information (41.9%, 96/227 vs 34.4%, 78/227; *P*=.001). Furthermore, RCT participants more often used the internet (96.9%, 220/227 vs 66.4%, 152/229; *P*<.001) (Table 1).

Emotional distress, as measured with the heiQ, was significantly lower among RCT participants compared with the score for paper respondents (Table 2), although the effect size was small (Cohen *d*=0.25). No significant differences were observed regarding other self-management skills between the RCT and paper groups. In addition, no significant differences were observed in the proportion of patients with psychological distress between RCT participants and paper respondents, although paper participants seemed to have higher mean scores. Furthermore, no differences were observed between RCT participants and paper respondents regarding satisfaction with overall information provision (Table 2).

**Table 2:** Differences in randomized controlled trial (RCT) outcome variables at baseline between RCT participants and paper respondents.

Outcome variable		RCT participants (N=227)	Paper respondents (N=229)	*P* value	Cohen *d*
Psychological distress (yes), n (%)		34 (15.0)	45 (19.7)	.18	
Psychological distress^a^, mean (SD)		6.5 (5.9)	7.5 (6.1)	.06	0.18
**Self-management skills^b^, mean (SD)**					
	Health-directed behavior	3.3 (0.6)	3.2 (0.6)	.12	0.14
	Positive and active engagement in life	3.2 (0.5)	3.1 (0.5)	.05	0.18
	Self-monitoring and insight	3.0 (0.4)	3.1 (0.4)	.62	0.05
	Constructive attitudes and approaches	3.3 (0.5)	3.3 (0.5)	.66	0.04
	Skill and technique acquisition	2.9 (0.5)	3.0 (0.5)	.33	0.09
	Social integration and support	3.2 (0.5)	3.2 (0.5)	.77	0.03
	Health services navigation	3.3 (0.4)	3.3 (0.4)	.54	0.06
	Emotional distress	1.8 (0.5)	1.9 (0.6)	**.**01	0.25
**Satisfaction with overall information provision, n (%)**				.29	
	Very unsatisfied	1 (0.4)	2 (0.9)		
	Unsatisfied	9 (4.0)	8 (3.5)		
	Neither	49 (21.6)	41 (17.9)		
	Satisfied	126 (55.5)	143 (62.4)		
	Very satisfied	41 (18.1)	27 (11.8)		

^a^Scale 0-42; a higher score indicates more psychological distress.

^b^Scale 1-4; higher scores indicate better status or self-management, except for emotional distress, in which higher scores indicate higher distress.

## Discussion

### Principal Findings

This reach analysis among RCT participants within a population-based sample showed a selective reach with an underrepresentation of older patients, women, and those with a medium to low level of education. In addition, our RCT participants may represent individuals with relatively better psychological well-being as scores for emotional distress were lower in this group.

Approximately a quarter of the population-based sample of patients with lymphoma and CLL were assessed to be not eligible for the study for various reasons (eg, deceased, severe psychopathology or cognitive impairment, a different diagnosis was ultimately made). Among the eligible patients who were invited to participate, 51% responded and completed a questionnaire, half of whom completed the web-based questionnaire and were enrolled in the RCT, resulting in a participation rate of 25%. This means that only one in four of all eligible patients actually participated in the RCT. This participation rate was lower compared with that of an RCT on the fully automated electronic health (eHealth) application Oncokompas that supports cancer survivors in their self-management (48%) [24]. Similar to the patients in our RCT, patients in the Oncokompas RCT were selected from the population-based NCR. However, cancer survivors in the Oncokompas RCT were first invited in an online survey study on supportive care and eHealth to assess internet use. Their participation rate was calculated as the number of RCT participants divided by the number of eligible respondents of the survey (access to the internet and email address). Thus, their group of eligible respondents was more selective compared with our sample. In our sample, only 82% of all respondents used the internet, and this percentage may be even lower among all patients invited to participate.

The results of the current study demonstrate that the RCT participants were younger, more often men, and more often actively treated compared to nonparticipants. Thus, the sample of RCT participants may not be representative of the target population. Therefore, even though the sample size reached the required number of patients [12], this sample may not be reliable for drawing conclusions about the target population. Furthermore, the effects of the intervention on the target population may be different from the effects that were found in the RCT sample [10,25].

These results also provide information about the response rate of observational research, which was 51%. This is comparable with response rates from other population-based studies on quality of life among lymphoma survivors in Germany (54.7%) [26] and the United States (54.8%) [27]. However, the current response rate is lower compared with that reported from earlier observational research within our study group at approximately 80%, despite similar patients and recruitment procedures [28]. This might be explained by the knowledge that the more information that is disclosed about the study—which is inherently more for an RCT than for observational research—the higher the proportion of nonrespondents [29]. Patients received abundant information along with the invitation, especially about participation in an RCT and randomization. The amount of information, as well as the knowledge of being randomized when completing a web-based questionnaire, may have deterred patients from participating. In addition, the type of intervention may have influenced the participation rate, as the majority of patients did not have problems with emotional adjustment to having cancer and therefore may have been less interested in a self-management intervention. Another explanation may be related to the fact that participation and response rates for health-related research have been declining over the past several years [30,31], and potential participants are faced with an increasing number of requests to participate in studies. This may result in patients refusing to participate in all studies [32].

We further compared the characteristics of RCT participants with those of paper respondents. RCT participants, who completed the web-based questionnaire, were younger, more often men, and more highly educated than the paper respondents, which are similar to the characteristics from previous observational studies within our study group [33,34]. Highly educated patients more often display prosocial behavior than patients with lower levels of education, and therefore the former group may be more likely to participate in an RCT for altruistic reasons [35]. In addition to differences in sociodemographic characteristics, RCT participants reported lower scores related to neuroticism and higher scores related to openness than paper respondents. In addition, information preferences slightly differed between RCT and paper respondents, as RCT participants more often wanted to receive all available information on their disease. RCT participants also more often reported using the internet. To complete a web-based questionnaire, and subsequently be enrolled in a web-based self-management intervention RCT, patients must not only be able to use a computer but also be sufficiently skilled in browsing the internet [33]. Although there seems to be a trend of older individuals becoming more active online [33,36], there is still a subgroup of patients who do not use the internet and thus have no access to a web-based questionnaire or internet-based intervention.

Despite these various differences between RCT participants and paper participants, baseline scores on self-management skills, satisfaction with information provision, and psychological distress appeared to be comparable between these groups, although scores for emotional distress were slightly lower among RCT participants.

### Strengths

The strengths of this study include its unique setting. As patients were recruited from the population-based NCR, we had information on sociodemographic and clinical characteristics of both RCT participants and nonparticipants. In addition, as the RCT was embedded in the PROFILES registry, we were able to assess differences between RCT participants and paper respondents on sociodemographic and clinical characteristics, in addition to personality traits, information preferences, and baseline psychological distress, self-management skills, and satisfaction with information provision (ie, RCT outcome variables). This information provided the opportunity to determine both the reach and generalizability of the RCT sample.

### Limitations

The current study has some limitations. Although information regarding sociodemographic and clinical characteristics of nonparticipants was available, we did not have information about nonparticipants’ reasons for declining participation or their physical and psychological health. Therefore, it remains unclear whether the physical and psychological health of RCT participants is similar to that of nonparticipants. In a previous study that assessed the generalizability of the results of observational research among cancer survivors by comparing characteristics of participants and nonparticipants, sensitivity analysis demonstrated that quality of life might be lower among nonparticipants [34]. As RCT participants may have a systematically higher quality of life or report fewer symptoms compared with nonparticipants, observed outcomes may represent a group of healthier patients with better outcomes. This may lead to circumspection in generalizing the results of an RCT to the target population. It is important to keep this in mind when interpreting RCT results that may only represent a minority of the target population.

### Conclusions

The participation rate in the RCT was 25%. RCT participants may be not representative of the target population owing to different sociodemographic and clinical characteristics. RCT results should be considered with caution, as RCT participants represent a minority of the target population, and may actually be those least in need of the intervention.

## Abbreviations

CLL: chronic lymphocytic leukemia
eHealth: electronic health
HeiQ: Health Education Impact Questionnaire
LIVE: Lymphoma InterVEntion
NCR: Netherlands Cancer Registry
PROFILES: Patient Reported Outcomes Following Initial treatment and Long term Evaluation of Survivorship
RCT: randomized controlled trial
